# Blue Rubber Bleb Nevus Syndrome as a Cause of Lower Digestive Bleeding

**DOI:** 10.1155/2014/683684

**Published:** 2014-02-05

**Authors:** Carlos Augusto Real Martinez, Murilo Rocha Rodrigues, Daniela Tiemi Sato, Paulo Pedroso Silveira Júnior, Rafael Fernandes Gama, Christian Bornia Mattavelli, José Aires Pereira

**Affiliations:** ^1^Universidade São Francisco (USF), 12914-450 Bragança Paulista, SP, Brazil; ^2^Medical School of Universidade São Francisco (USF), 12914-450 Bragança Paulista, SP, Brazil

## Abstract

*Introduction*. Blue rubber bleb nevus syndrome is a rare disorder that is characterized by multiple recurrent vascular malformations that involve the skin and the gastrointestinal tract. The disease can present chronic anemia and severe episodes of gastrointestinal bleeding. *Case Report*. A 41-year-old man was admitted with recurrent episodes of lower gastrointestinal bleeding and anemia that had worsened over the last 3 months. The physical examination showed soft, diffuse, compressible, bluish nodules on all of the skin surfaces of the body. A biopsy from one of these skin lesions allowed a histological diagnosis of cavernous hemangioma. He submitted to a colonoscopy, which showed hemorrhoids and a plane vascular lesion mainly located on the right colon with recent signs of bleeding; this lesion was treated by local excision and sclerosis. The pathological study of the colon specimens also reflected the presence of cavernous hemangioma. The cutaneous hemangiomas and the presence of colonic venous malformations were compatible with blue rubber bleb nevus syndrome. The patient presented a favorable follow-up with clinical control of the anemia and without relapse of the gastrointestinal bleeding two years after the procedure. *Conclusion*. Although rarely diagnosed, blue rubber bleb nevus syndrome may be responsible for lower digestive bleeding.

## 1. Introduction

Blue rubber nevus syndrome (BRNS), also known as Bean Syndrome, is a rare angiomatosis condition with an estimated incidence of 1 : 14,000 births [1, 2]. This syndrome is characterized by the association of cutaneous and gastrointestinal venous malformations with iron deficiency anemia or gastrointestinal bleeding [3]. The disease was described by Gascoyen (1860) [4], but Bean differentiated this type of angiomatosis from other vascular diseases of the skin in 1958 [5]. BRNS can affect many organs and, since its initial description, it is estimated that there have been fewer than 200 cases published to date [6, 7]. This disease affects both adults and children of both sexes but is rare among black subjects [8]. In most cases, it is not possible to identify a family history, although a dominant pattern of inheritance has been reported [9].

The purpose of this paper is to describe a case of BRNS with cutaneous and colon involvement that evolved from lower gastrointestinal bleeding, the diagnosis of which was confirmed by histopathological study.

## 2. Case Report

**Figure 1:**
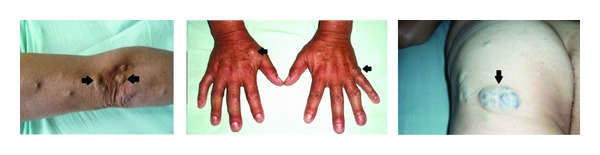
Formation of bluish hemangiomas on the arms, forearm, hands, and thighs (arrows).

A 41-year-old black male was admitted for the investigation of recurrent episodes of lower gastrointestinal bleeding over the previous 10 years that had become more frequent over the last three months. Since childhood, he had undergone repeated treatments of oral iron supplementation for anemia that was difficult to clinically control. He had received blood transfusions at two instances to control the severe anemia. The patient reported that, since childhood, he had blue spots, similar to varicose veins, distributed in his hands, arms, legs, and trunk that increased in size and number with his age. The lesions had varying diameters (1 cm to 12 cm) and were usually regularly raised mushrooms, resembling small bubbles of blue coloration (Figure 1). One of these skin lesions was located at the elbow and had demonstrated several episodes of bleeding after trauma; it had been excised and sent for examination. The histopathological examination of this lesion confirmed the diagnosis of cutaneous hemangioma. Upon physical examination, the patient was in a good general condition with severe mucocutaneous pallor. The examination of the skin on the body's surface showed numerous injuries resembling raised mushrooms with soft consistency and depressive compression that most often involved the trunk and upper limbs. The red blood cell counts showed severe anemia (Hemoglobin 6.1 g/dL) and hypochromic microcytosis with the presence of 4% reticulocytes. The serum iron level was 18 mg/dL. Upon digital rectal examination, we found blood on the glove without palpable masses.

**Figure 2:**
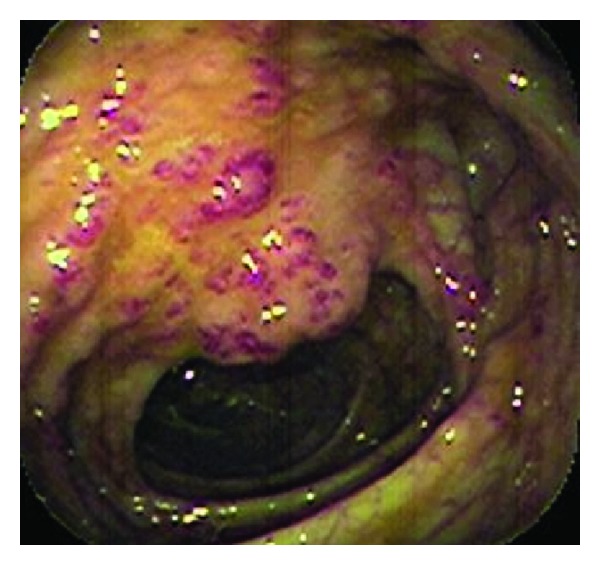
Colonoscopy showing numerous vascular malformations located in the surface of the colon mucosa.

**Figure 3:**
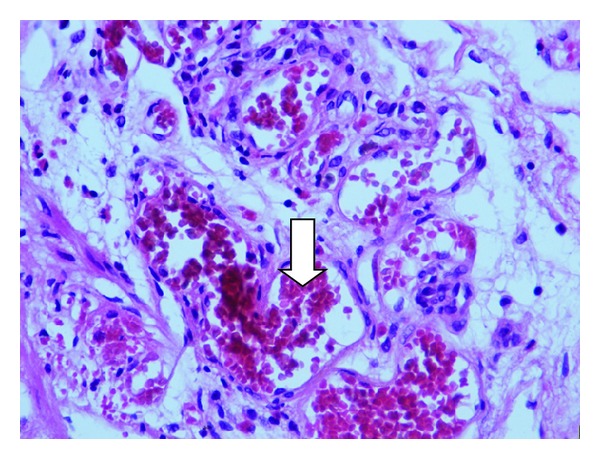
Colon biopsy showing vascular malformation with dilatation and the congestion of the blood capillaries of the submucosa (white arrow). (HE ×400).

To clarify the origin of the lower digestive bleeding, the patient underwent a colonoscopy that identified, in addition to hemorrhoidal disease of the III degree, numerous venous malformations measuring 1.5 × 2.0 cm that were scattered throughout the mucosa of the colon and rectum (Figure 2). One of these lesions showed signs of recent bleeding after being removed with snare cautery. Histopathological examination revealed numerous dilated blood vessels in the lesion with signs of recent hemorrhage located mainly in the submucosal layer (Figure 3). The presence of hemangiomas of the colon associated with cutaneous venous malformations allowed the diagnosis of BRNS. The hemorrhoidal disease was treated by two sessions of rubber band ligation, which were performed uneventfully. Currently, the patient is healthy and has not presented with new episodes of gastrointestinal bleeding; his anemia is well-controlled two years after the described procedures.

## 3. Discussion

The BRNS was most likely initially observed by Camilleri et al. in 1948 [2]. After a century, Bean [3] became the first author to use the term to describe this rare congenital disorder, which is characterized by vascular malformations that affect the skin and the viscera of the gastrointestinal tract. In the original article, the author described three types of cutaneous vascular lesions that are commonly observed in the disease: (1) bluish lesions with soft consistency, smooth or wrinkled, similar to blebs filled with blood that are easily depressible and quickly reperfused when the pressure is relieved; (2) disfiguring lesions, vascular malformations, and cavernous large lesions that can compress vital structures; and (3) macular lesions with irregular blue staining diffusely scattered throughout the body [3, 7]. The literature review shows that, since the initial description, fewer than an estimated 200 cases of BRNS have been published to date [4, 7].

BRNS is characterized by the appearance of venous malformations of the skin that are present from birth or childhood and increase in size and number with increasing age [10, 11]. The majority of the lesions are asymptomatic, but some may be painful upon compression. When located on the skin, they rarely bleed unless they are located within a topography that may suffer injuries, as occurred in the patient here described. BRNS is usually described in sporadic cases, but there may be autosomal dominant trait that can be inherited by different affected family members [12]. Recent genetic studies have identified *locus* on chromosome 9 that is responsible for the development of venous malformations [13]. As the pathogenesis of BRNS is not yet fully understood, new research is being conducted with the intention of elucidating its pathogenesis and enabling more effective genetic counseling.

The involvement of the gastrointestinal tract arises during adulthood, when the patient begins to exhibit anemia and occult blood in the stool or gastrointestinal bleeding. Venous malformations can affect any segment of the digestive tract from the oral mucosa to the anal canal, but in these cases, they are located most frequently in the small intestine and colon [14, 15]. In contrast to skin lesions, gastrointestinal vascular malformations tend to bleed more easily, resulting from a loss of insensitive, bloody stools and severe lower gastrointestinal bleeding that requires blood transfusions. When the bleeding is insidious, it can lead to anemia of the chronic iron deficiency type, requiring constant replenishment of iron or, less often, blood replacement. Rarely, patients may experience recurrent abdominal pain and framed sub-occlusion episodes due to intestinal intussusception [7, 16]. Early diagnosis is important for the potential possibility of severe gastrointestinal bleeding. A colonoscopy is the ideal exam for the diagnosis of the venous malformations located in the colon. However, when the small bowel angiomatosis is compromised, it is often necessary to perform CT enterography, magnetic resonance imaging, double-balloon enteroscopy or endoscopic wireless capsule [17–19]. Selective arteriography and scintigraphy are useful in identifying the source of the bleeding in cases where the patient has active bleeding [20]. In the patient in this report, the colonoscopy identified multiple venous malformations in the colon but was not able to find any venous malformations in the 15 cm ileal segment that was investigated.

BRNS has also been described in association with other vascular malformations located in the nasopharynx, eyes, thyroid, parotid, central nervous system, musculoskeletal system, pleura, peritoneum, pericardium, mesentery, lungs, kidneys, liver, spleen, penis, vulva, and bladder [21]. Systemic complications, such as thrombosis and the calcification of vascular lesions, may also occur in these patients. Some patients may present consumption coagulopathy, thrombocytopenia, or chronic lymphocytic leukemia [6]. In cases where the intestinal bleeding represents a greater proportion, hemodynamic instability can occur, with varying degrees of shock and even death [9].

Noncurative treatments also exist for BRNS. The cutaneous lesions in most cases do not require surgical removal, unless aesthetic damage is present or they impose functional restrictions. The treatment of skin lesions is limited to surgical excisions or laser ablations when they become symptomatic or when they are located in areas subject to trauma (pleated elbow, knee, buttocks, palms, and fingers), as noted in the patient of this report. The treatment for most patients is clinical and consists of controlling the anemia via constant iron replenishment and blood transfusions in patients with severe anemia. Antiangiogenic substances, corticosteroids, propranolol, and interferon-*α* have been used based on evidence that vascular malformations may regress, similar to what occurs with hemangiomas during infancy [22, 23]. The use of sirolimus drugs with potent antiangiogenic activities has recently been proposed to successfully treat BRNS [21]. Similarly, octreotide also appears to have therapeutic effects for cases with gastrointestinal bleeding [7]. However, there is insufficient evidence in the literature regarding the duration of the therapeutic benefits of any of these drugs proposed for the clinical treatment of BRNS. Minimally invasive treatment by photocoagulation using laser or argon plasma and the endoscopic removal of the lesions by a variety of different techniques have also been used in a small proportion of patients, but the effects of these strategies seldom prevent the recurrence of bleeding [24, 25].

The treatment of gastrointestinal lesions varies according to its length, position, and evolution. In patients with a small number of lesions or diffuse lesions that evolve with anemia but without gastrointestinal bleeding, the obvious clinical treatment seems to be the recommended practice. In those patients who have severe gastrointestinal bleeding or anemia, the main measures proposed to be less invasive include unwieldy clinical surgical excisions, endoscopic sclerotherapy, rubber band ligation, and laser therapies [26]. A total colectomy or segmental resection of the small intestine is reserved for cases where the disease is extremely localized or in cases of bleeding refractory to conservative measures [27]. It should be emphasized that the surgical resection of these lesions is rarely successful, due to the extensive nature of the diffuse venous malformations along the gastrointestinal tract. The patient in this report was treated by endoscopic excision and cauterization of the vascular malformation in the colon after showing signs of bleeding, and he was later treated by rubber band ligation of the hemorrhoids' vessels. At present, two years after the completion of both procedures, there has been no recurrence of the bleeding.

Considering that this is a rarely reported disease, this report stresses the importance of the diagnosis of BRNS among young patients with a history of chronic anemia and urges clinicians to submit pictures of gastrointestinal hemorrhages associated with cutaneous vascular malformations for analysis. When a diagnosis of BRNS is made, the patient should be informed about the chronic nature of this disease, which imposes the need for regular follow-up.
